# Supplementary material for: SeqForge: a scalable platform for alignment-based searches, motif detection, and sequence curation across meta/genomic datasets
Source: BMC Bioinformatics. 2025 Nov 18;26:280. doi: 10.1186/s12859-025-06297-9 (PMC12625553; doi:10.1186/s12859-025-06297-9)
Supplement: Supplementary file 1 — Supplementary Material 1 [file 12859_2025_6297_MOESM1_ESM.docx]

SeqForge: A scalable platform for alignment-based searches, motif detection, and sequence curation across meta/genomic datasets

Elijah R. Bring Horvath^1,*^ and Jaclyn M. Winter^1,*^

^1^Department of Pharmacology and Toxicology, University of Utah, Salt Lake City, Utah, 84112, United States

* To whom correspondence should be addressed: Tel: [+1 801-581-6353]; Email: [jaclyn.winter@utah.edu]. Correspondence may also be addressed to Elijah R. Bring Horvath; Email: [eli.bringhorvath@pharm.utah.edu].

| **Table of Contents** | |
| --- | --- |
|  |  |
| Extended Methods | 2 |
| Tables S1-S3: Information on genomes used in this study. | 3 |
| Table S4: Erythromycin motif mining output | 4 |
| Figure S1: Aligned erythromycin AT domains | 6 |
| Figure S2: Aligned erythromycin KS domains | 7 |
| Figure S3: Aligned erythromycin KR domains | 8 |
| Table S5: Predicted functions of atpenin B biosynthetic genes | 9 |
| Table S6: *Penicillium* genomes meeting ApnU tblastn inclusion thresholds | 10 |
| Figure S4: Aligned ApnU sequences | 11 |
| Table S7: SeqForge FASTA-metrics module compared to QUAST assembly metrics output | 12 |
| References | 14 |

**Extended Methods**

Augustus Penicillium Model

The genome and predicted coding sequences of the *Penicillium chrysogenum* strain IBT 35668 (Accession: GCA_028827035.1) was downloaded from NCBI and used as a reference for model training. Training and coding sequence (CDS) prediction was carried out using Braker2 v2.1.6 [1], BUSCO v5.4.3 (<https://github.com/metashot/busco>), RepeatModeler v1.0.8 [2], and AUGUSTUS v3.5.0 [3]. RepeatModeler was run using default settings and returned an empty consensus file; upon further investigation, the *P. chrysogenum* IBT 35668 genome had already undergone soft masking. BRAKER2 was executed using the following command string: braker.pl --genome ./genome/GCA_028827035_1.fna --prot_seq ./proteins/GCA_028827035_1_proteins.faa --softmasking --gff3 --cores 32. BUSCO was run as follows: busco -i braker/augustus.hints.aa -l eurotiomycetes_odb10 -m proteins -o busco_penicillium -c 16, and returned the following results: C:98.5% (S: 97.6%, D: 0.9%) F: 0.8%, M: 0.7%, n: 3546. Where: C = 3493 complete BUSCOS, S = 3462 complete and single-copy BUSCOS, D = 31 complete and duplicated BUSCOS, F = 27 fragmented BUSCOS, and M = 26 missing BUSCOS. CDS predictions were performed with AUGUSTUS using default settings.

Tables S1–S3: Information on the *E. coli*, *Streptomyces,* and *Penicillium* genomes used in this study. Due to their size, these tables are available as separate downloadable files at https://figshare.com/articles/software/SeqForge_Software_and_Supplementary_Tables/29897087.

Table S4. Raw output of key motif mining in erythromycin acyltransferase (AT), ketosynthase (KT), and ketoreductase (KR) domains. Motif 5 (HXFH) returned no hits, and those columns were therefore removed. A single off-target hit for HXSH was returned but discarded as it overlapped with the appropriate YXXH motif.

| genome | query | sseqid | sstart | send | motif_1 | motif_1_pattern | motif_2 | motif_2_pattern | motif_3 | motif_3_pattern | motif_4 | motif_4_pattern | motif_6 | motif_6_pattern | motif_7 | motif_7_pattern | motif_8 | motif_8_ pattern | match_start | match_end |
| --- | --- | --- | --- | --- | --- | --- | --- | --- | --- | --- | --- | --- | --- | --- | --- | --- | --- | --- | --- | --- |
| erythromycin_BGC | AT2 | erythromycin_S_erythraea_13 | 2016 | 2311 | RVDVLQ | RVXXXQ |  |  |  |  |  |  |  |  |  |  |  |  | 2072 | 2077 |
| erythromycin_BGC | AT2 | erythromycin_S_erythraea_11 | 1048 | 1348 | RVDVVQ | RVXXXQ |  |  |  |  |  |  |  |  |  |  |  |  | 1110 | 1115 |
| erythromycin_BGC | AT2 | erythromycin_S_erythraea_11 | 2526 | 2822 | RVDVVQ | RVXXXQ |  |  |  |  |  |  |  |  |  |  |  |  | 2581 | 2586 |
| erythromycin_BGC | AT2 | erythromycin_S_erythraea_13 | 562 | 832 | RVDVVQ | RVXXXQ |  |  |  |  |  |  |  |  |  |  |  |  | 618 | 623 |
| erythromycin_BGC | AT2 | erythromycin_S_erythraea_14 | 556 | 830 | RVDVVQ | RVXXXQ |  |  |  |  |  |  |  |  |  |  |  |  | 609 | 614 |
| erythromycin_BGC | AT2 | erythromycin_S_erythraea_14 | 2019 | 2309 | RVDVVQ | RVXXXQ |  |  |  |  |  |  |  |  |  |  |  |  | 2074 | 2079 |
| erythromycin_BGC | AT2 | erythromycin_S_erythraea_11 | 73 | 366 | RVEVVQ | RVXXXQ |  |  |  |  |  |  |  |  |  |  |  |  | 126 | 131 |
| erythromycin_BGC | KR1 | erythromycin_S_erythraea_11 | 1628 | 1805 |  |  |  |  |  |  |  |  |  |  | HAAATLDDG | HXAXXLDDX |  |  | 1713 | 1721 |
| erythromycin_BGC | KR1 | erythromycin_S_erythraea_11 | 1628 | 1805 |  |  |  |  |  |  |  |  |  |  |  |  | SSFASAFGAPGLGGYAP | SSXXXXXXXXXXXXYXX | 1761 | 1777 |
| erythromycin_BGC | KR1 | erythromycin_S_erythraea_11 | 3073 | 3249 |  |  |  |  |  |  |  |  |  |  |  |  | SSGAGVWGSARQGAYAA | SSXXXXXXXXXXXXYXX | 3205 | 3221 |
| erythromycin_BGC | KR1 | erythromycin_S_erythraea_14 | 1116 | 1292 |  |  |  |  |  |  |  |  |  |  |  |  | SSNAGVWGSPGLASYAA | SSXXXXXXXXXXXXYXX | 1248 | 1264 |
| erythromycin_BGC | KR1 | erythromycin_S_erythraea_14 | 2556 | 2730 |  |  |  |  |  |  |  |  |  |  |  |  | SSGAGVWGSANLGAYSA | SSXXXXXXXXXXXXYXX | 2686 | 2702 |
| erythromycin_BGC | KR1 | erythromycin_S_erythraea_13 | 3141 | 3317 |  |  |  |  |  |  |  |  |  |  |  |  | SSAASVLAGPGQGVYAA | SSXXXXXXXXXXXXYXX | 3273 | 3289 |
| erythromycin_BGC | KR1 | erythromycin_S_erythraea_13 | 1132 | 1297 |  |  |  |  |  |  |  |  |  |  |  |  | SSVAGIWGGAGMAAYAA | SSXXXXXXXXXXXXYXX | 1253 | 1269 |
| erythromycin_BGC | AT2 | erythromycin_S_erythraea_11 | 1048 | 1348 |  |  | GHSQGE | GHXXGE |  |  |  |  |  |  |  |  |  |  | 1141 | 1146 |
| erythromycin_BGC | AT2 | erythromycin_S_erythraea_11 | 1048 | 1348 |  |  |  |  | YASH | YXXH |  |  |  |  |  |  |  |  | 1240 | 1243 |
| erythromycin_BGC | AT2 | erythromycin_S_erythraea_11 | 1048 | 1348 |  |  |  |  |  |  | HSSH | HXSH |  |  |  |  |  |  | 1243 | 1246 |
| erythromycin_BGC | AT2 | erythromycin_S_erythraea_11 | 2526 | 2822 |  |  | GHSQGE | GHXXGE |  |  |  |  |  |  |  |  |  |  | 2612 | 2617 |
| erythromycin_BGC | AT2 | erythromycin_S_erythraea_11 | 2526 | 2822 |  |  |  |  | YASH | YXXH |  |  |  |  |  |  |  |  | 2716 | 2719 |
| erythromycin_BGC | AT2 | erythromycin_S_erythraea_11 | 73 | 366 |  |  | GHSIGE | GHXXGE |  |  |  |  |  |  |  |  |  |  | 157 | 162 |
| erythromycin_BGC | AT2 | erythromycin_S_erythraea_13 | 2016 | 2311 |  |  | GHSQGE | GHXXGE |  |  |  |  |  |  |  |  |  |  | 2103 | 2108 |

Table S4 continued.

| genome | query | sseqid | sstart | send | motif_1 | motif_1_pattern | motif_2 | motif_2_pattern | motif_3 | motif_3_pattern | motif_4 | motif_4_pattern | motif_6 | motif_6_pattern | motif_7 | motif_7_pattern | motif_8 | motif_8_pattern | match_start | match_end |
| --- | --- | --- | --- | --- | --- | --- | --- | --- | --- | --- | --- | --- | --- | --- | --- | --- | --- | --- | --- | --- |
| erythromycin_BGC | AT2 | erythromycin_S_erythraea_13 | 2016 | 2311 |  |  |  |  | YASH | YXXH |  |  |  |  |  |  |  |  | 2205 | 2208 |
| erythromycin_BGC | AT2 | erythromycin_S_erythraea_13 | 562 | 832 |  |  | GHSQGE | GHXXGE |  |  |  |  |  |  |  |  |  |  | 649 | 654 |
| erythromycin_BGC | AT2 | erythromycin_S_erythraea_13 | 562 | 832 |  |  |  |  | YASH | YXXH |  |  |  |  |  |  |  |  | 751 | 754 |
| erythromycin_BGC | AT2 | erythromycin_S_erythraea_14 | 556 | 830 |  |  | GHSQGE | GHXXGE |  |  |  |  |  |  |  |  |  |  | 640 | 645 |
| erythromycin_BGC | AT2 | erythromycin_S_erythraea_14 | 556 | 830 |  |  |  |  | YASH | YXXH |  |  |  |  |  |  |  |  | 742 | 745 |
| erythromycin_BGC | AT2 | erythromycin_S_erythraea_14 | 2019 | 2309 |  |  | GHSQGE | GHXXGE |  |  |  |  |  |  |  |  |  |  | 2105 | 2110 |
| erythromycin_BGC | AT2 | erythromycin_S_erythraea_14 | 2019 | 2309 |  |  |  |  | YASH | YXXH |  |  |  |  |  |  |  |  | 2207 | 2210 |
| erythromycin_BGC | KS | erythromycin_S_erythraea_11 | 522 | 945 |  |  |  |  |  |  |  |  | TACSSS | TAXSSX |  |  |  |  | 690 | 695 |
| erythromycin_BGC | KS | erythromycin_S_erythraea_11 | 1997 | 2416 |  |  |  |  |  |  |  |  | TACSSS | TAXSSX |  |  |  |  | 2162 | 2167 |
| erythromycin_BGC | KS | erythromycin_S_erythraea_13 | 1491 | 1910 |  |  |  |  |  |  |  |  | TACSSS | TAXSSX |  |  |  |  | 1659 | 1664 |
| erythromycin_BGC | KS | erythromycin_S_erythraea_13 | 33 | 457 |  |  |  |  |  |  |  |  | TACSSS | TAXSSX |  |  |  |  | 200 | 205 |
| erythromycin_BGC | KS | erythromycin_S_erythraea_14 | 41 | 450 |  |  |  |  |  |  |  |  | TACSSG | TAXSSX |  |  |  |  | 197 | 202 |
| erythromycin_BGC | KS | erythromycin_S_erythraea_14 | 1488 | 1909 |  |  |  |  |  |  |  |  | TACSSS | TAXSSX |  |  |  |  | 1655 | 1660 |

Figure S1. Aligned acyltransferase (AT) domains from the erythromycin biosynthetic gene cluster in *Saccharopolyspora erythraea* (MIBiG accession BGC0000055). ATL: acyltransferase loading domain. Key motifs are outlined with black dashed lines.

Figure S2. Aligned ketosynthase (KS) domains from the erythromycin biosynthetic gene cluster in *Saccharopolyspora erythraea* (MIBiG accession BGC0000055). Active site motif is outlined with a black dashed line.

Figure S3. Aligned ketoreductase (KR) domains from the erythromycin biosynthetic gene cluster in *Saccharopolyspora erythraea* (MIBiG accession BGC0000055). Key motifs are outlined with black dashed lines.

Table S5. Predicted functions of atpenin B biosynthetic genes. *function has been biochemically characterized, but NCBI BLAST annotation has not been updated.

| **Gene** | **Function/Predicted Function** | **Identity (%)** | **Query Coverage (%)** | **NCBI Accession** |
| --- | --- | --- | --- | --- |
| *apnU* | Cu(II)-dependent halogenase* | 100 | 100 | XP_049973506.1 |
| *orf2* | Hypothetical | 100 | 100 | EPS34229.1 |
| *apnC* | FAD-dependent monooxygenase | 100 | 99.3 | XP_049973504.1 |
| *orf1* | Hypothetical | 100 | 100 | EPS34231.1 |
| *apnB* | Hypothetical | 100 | 99.76 | XP49973502.1 |
| *apnH* | Hypothetical | 100 | 100 | EPS34233.1 |
| *apnA* | Polyketide synthase | 100 | 98.97 | XP049973499.1 |
| *apnG* | Cytochrome monooxygenase | 100 | 100 | XP049973498.1 |
| *apnF* | FAD-dependent monooxygenase | 100 | 99.83 | XP049973497.1 |
| *apnE* | Trans-enoyl reductase | 100 | 99.72 | XP049973496.1 |
| *apnD* | Cytochrome monooxygenase | 100 | 99.58 | XP_049973495.1 |

Table S6. *Penicillium* genomes meeting ApnU query inclusion thresholds of % identity ≥ 80% and query coverage ≥ 70%. Table represents all_filtered_results.csv generated from SeqForge’s query module.

| **qseqid** | **sseqid** | **database** | **query_file_name** | **pident** | **query_ coverage** | **evalue** | **bitscore** | **length** | **mismatch** | **gapopen** | **qstart** | **qend** | **sstart** | **send** | **qlen** | **sframe** |
| --- | --- | --- | --- | --- | --- | --- | --- | --- | --- | --- | --- | --- | --- | --- | --- | --- |
| ApnU | JARG01000001.1 | GCA_002072455_1_Pexp1_0_genomic | ApnU | 84.775 | 99.59 | 9.69E-162 | 495 | 289 | 0 | 2 | 1 | 245 | 6402133 | 6401267 | 245 | -3 |
| ApnU | SDBR01000018.1 | GCA_004521935_1_ASM452193v1_genomic | ApnU | 84.775 | 99.59 | 9.25E-162 | 495 | 289 | 0 | 2 | 1 | 245 | 324004 | 323138 | 245 | -2 |
| ApnU | CP093053.1 | GCA_022570495_1_ASM2257049v1_genomic | ApnU | 84.775 | 99.59 | 9.51E-162 | 495 | 289 | 0 | 2 | 1 | 245 | 2315783 | 2316649 | 245 | 2 |
| ApnU | KB644415.1 | GCA_000346795_1_pde_v1_0_genomic | ApnU | 84.775 | 99.59 | 9.36E-162 | 495 | 289 | 0 | 2 | 1 | 245 | 3584465 | 3583599 | 245 | -2 |
| ApnU | JASKYK010000001.1 | GCA_030378375_1_ASM3037837v1_genomic | ApnU | 84.775 | 99.59 | 9.55E-162 | 495 | 289 | 0 | 2 | 1 | 245 | 3522649 | 3521783 | 245 | -2 |
| ApnU | CM041256.1 | GCA_001723175_3_ASM172317v3_genomic | ApnU | 84.775 | 99.59 | 9.55E-162 | 495 | 289 | 0 | 2 | 1 | 245 | 2334210 | 2335076 | 245 | 3 |
| ApnU | CP088335.1 | GCA_021133555_1_ASM2113355v1_genomic | ApnU | 84.775 | 99.59 | 9.47E-162 | 495 | 289 | 0 | 2 | 1 | 245 | 3546551 | 3545685 | 245 | -1 |
| ApnU | KB908904.1 | GCA_000383025_1_pdt_v1_0_genomic | ApnU | 84.775 | 99.59 | 9.52E-162 | 495 | 289 | 0 | 2 | 1 | 245 | 3534979 | 3534113 | 245 | -2 |
| ApnU | SZWD01000001.1 | GCA_005546515_1_SGAir0226_genomic | ApnU | 84.775 | 99.59 | 5.70E-162 | 496 | 289 | 0 | 2 | 1 | 245 | 1257011 | 1257877 | 245 | 2 |
| ApnU | JAMABO010000024.1 | GCA_023624835_1_ASM2362483v1_genomic | ApnU | 84.775 | 99.59 | 9.24E-162 | 495 | 289 | 0 | 2 | 1 | 245 | 195075 | 194209 | 245 | -1 |
| ApnU | JAQJAE010000006.1 | GCA_028827395_1_ASM2882739v1_genomic | ApnU | 81.609 | 70.61 | 7.10E-94 | 301 | 174 | 27 | 1 | 72 | 245 | 3429489 | 3428983 | 245 | -3 |
| ApnU | JAMABN010000061.1 | GCA_023624855_1_ASM2362485v1_genomic | ApnU | 84.775 | 99.59 | 9.34E-162 | 495 | 289 | 0 | 2 | 1 | 245 | 54354 | 53488 | 245 | -3 |
| ApnU | JAPQKL010000007.1 | GCA_028826915_1_ASM2882691v1_genomic | ApnU | 82.041 | 90.61 | 1.76E-137 | 426 | 245 | 22 | 1 | 23 | 245 | 183921 | 184655 | 245 | 3 |
| ApnU | SGAW01000018.1 | GCA_004153425_1_ASM415342v1_genomic | ApnU | 84.775 | 99.59 | 9.25E-162 | 495 | 289 | 0 | 2 | 1 | 245 | 324004 | 323138 | 245 | -2 |

Figure S4. Alignment of the copper-dependent halogenase, ApnU, from the atpenin B biosynthetic gene cluster (MIBiG accession BGC0002067) against putative homologs identified in publicly available *Penicillium* genomes. Key HXXHC motifs are outlined with black dashed lines.

Table S7. SeqForge FASTA-metrics module compared to QUAST assembly metrics output.

| **QUAST** | ***E. coli*** | ***E. coli*** | ***E. coli*** | ***Streptomyces*** | ***Streptomyces*** | ***Streptomyces*** |
| --- | --- | --- | --- | --- | --- | --- |
| Assembly | GCA_037575275_1 | GCA_043933725_1 | GCA_047199145_1 | GCA_049606955_1 | GCA_050871035_1 | GCA_050953795_1 |
| # contigs (>= 0 bp) | 8 | 40 | 1 | 5 | 1 | 9 |
| # contigs (>= 1000 bp) | 8 | 11 | 1 | 5 | 1 | 9 |
| # contigs (>= 5000 bp) | 4 | 9 | 1 | 5 | 1 | 8 |
| # contigs (>= 10000 bp) | 3 | 7 | 1 | 4 | 1 | 7 |
| # contigs (>= 25000 bp) | 3 | 7 | 1 | 4 | 1 | 4 |
| # contigs (>= 50000 bp) | 3 | 4 | 1 | 4 | 1 | 3 |
| Total length (>= 0 bp) | 5711995 | 5506486 | 4593097 | 8512991 | 7609140 | 8175220 |
| Total length (>= 1000 bp) | 5711995 | 5493925 | 4593097 | 8512991 | 7609140 | 8175220 |
| Total length (>= 5000 bp) | 5699448 | 5490004 | 4593097 | 8512991 | 7609140 | 8171759 |
| Total length (>= 10000 bp) | 5691234 | 5478789 | 4593097 | 8505710 | 7609140 | 8162927 |
| Total length (>= 25000 bp) | 5691234 | 5478789 | 4593097 | 8505710 | 7609140 | 8116727 |
| Total length (>= 50000 bp) | 5691234 | 5372387 | 4593097 | 8505710 | 7609140 | 8075404 |
| # contigs | 8 | 22 | 1 | 5 | 1 | 9 |
| Largest contig | 5486274 | 5059890 | 4593097 | 6072140 | 7609140 | 7889737 |
| Total length | 5711995 | 5500528 | 4593097 | 8512991 | 7609140 | 8175220 |
| GC (%) | 50.5 | 50.52 | 50.85 | 73.69 | 72.03 | 72.03 |
| N50 | 5486274 | 5059890 | 4593097 | 6072140 | 7609140 | 7889737 |
| N90 | 5486274 | 5059890 | 4593097 | 2010573 | 7609140 | 7889737 |
| auN | 5273174.8 | 4662023.4 | 4593097 | 4820822.2 | 7609140 | 7617090.2 |
| L50 | 1 | 1 | 1 | 1 | 1 | 1 |
| L90 | 1 | 1 | 1 | 2 | 1 | 1 |
| # N's per 100 kbp | 0 | 0 | 0 | 0 | 1.58 | 0 |
|  |  |  |  |  |  |  |
| **SeqForge** | ***E. coli*** | ***E. coli*** | ***E. coli*** | ***Streptomyces*** | ***Streptomyces*** | ***Streptomyces*** |
| Filename | GCA_037575275_1 | GCA_043933725_1 | GCA_047199145_1 | GCA_049606955_1 | GCA_050871035_1 | GCA_050953795_1 |
| Num_Contigs | 8 | 22 | 1 | 5 | 1 | 9 |
| Num_Contigs_≥0bp | 8 | 40 | 1 | 5 | 1 | 9 |
| Num_Contigs_≥1kb | 8 | 11 | 1 | 5 | 1 | 9 |
| Num_Contigs_≥10kb | 3 | 7 | 1 | 4 | 1 | 7 |
| Num_Contigs_≥50kb | 3 | 4 | 1 | 4 | 1 | 3 |
| Num_Contigs_≥100kb | 2 | 2 | 1 | 3 | 1 | 2 |
| Total_Length | 5711995 | 5500528 | 4593097 | 8512991 | 7609140 | 8175220 |
| Total_Length_≥1kb | 5711995 | 5493925 | 4593097 | 8512991 | 7609140 | 8175220 |
| Longest_Contig | 5486274 | 5059890 | 4593097 | 6072140 | 7609140 | 7889737 |
| Shortest_Contig | 1902 | 509 | 4593097 | 7281 | 7609140 | 3461 |
| GC_Content(%) | 50.5 | 50.52 | 50.85 | 73.69 | 72.03 | 72.03 |
| N_Count | 0 | 0 | 0 | 0 | 0 | 0 |
| auN | 5273175 | 4662023 | 4593097 | 4820822 | 7609140 | 7617090 |

Table S7 continued.

| **SeqForge** | ***E. coli*** | ***E. coli*** | ***E. coli*** | ***Streptomyces*** | ***Streptomyces*** | ***Streptomyces*** |
| --- | --- | --- | --- | --- | --- | --- |
| N50 | 5486274 | 5059890 | 4593097 | 6072140 | 7609140 | 7889737 |
| N90 | 5486274 | 5059890 | 4593097 | 2010573 | 7609140 | 7889737 |
| L50 | 1 | 1 | 1 | 1 | 1 | 1 |
| L90 | 1 | 1 | 1 | 2 | 1 | 1 |

1. Bruna T, Hoff KJ, Lomsadze A, Stanke M, Borodovsky M. Braker2: Automatic eukaryotic genome annotation with genemark-ep+ and augustus supported by a protein database. NAR Genom Bioinform. 2021;3(1):lqaa108.

2. Flynn JM, Hubley R, Goubert C, Rosen J, Clark AG, Feschotte C, et al. Repeatmodeler2 for automated genomic discovery of transposable element families. Proc Natl Acad Sci U S A. 2020;117(17):9451-7.

3. Stanke M, Diekhans M, Baertsch R, Haussler D. Using native and syntenically mapped cdna alignments to improve de novo gene finding. Bioinformatics. 2008;24(5):637-44.
